# AI-accelerated compressed sensing and deep learning reconstruction for colon MRI: prospective validation of image quality and preliminary assessment of staging accuracy versus contrast-enhanced CT

**DOI:** 10.1186/s13244-026-02445-8

**Published:** 2026-11-03

**Authors:** Zhen Guan, Nan Sun, Tian-Jing Chang, Xiao Zhang, Sha Liu, Zi-Ran Li, Jun Yang, Rui-Jia Sun, Xiao-Yan Zhang, Ying-Shi Sun

**Affiliations:** 1 Department of Radiology, Key Laboratory of Carcinogenesis and Translational Research (Ministry of Education/Beijing), Peking University Cancer Hospital and Institute, No. 52 Fu Cheng Road, Hai Dian District, 100142 Beijing, China; 2 Department of Radiology, Peking University Shougang Hospital, No.9 Jin Yuan Zhuang Road, Shi Jing Shan District, 100144 Beijing, China; 3 Shanghai United Imaging Healthcare Co., Ltd, No. 2258, Chengbei Road, Jiading District, 201807 Shanghai, China

**Keywords:** Diagnostic imaging, Artificial intelligence, Colorectal neoplasms, Magnetic resonance imaging, Computed tomography

## Abstract

**Objectives:**

To prospectively validate an AI-accelerated MRI protocol combining L₁-regularized compressed sensing and deep learning reconstruction for colon cancer staging, compared with contrast-enhanced CT (CECT).

**Materials and methods:**

This single-center study (August 2022–April 2024) enrolled 132 patients with pathologically confirmed colon cancer who underwent both 3.0-T non-contrast MRI and CECT within 1 week. Patients unable to complete an MRI were excluded. MRI sequences included T1- and T2-weighted imaging and diffusion-weighted imaging. Image quality was assessed using a 4-point Likert scale, alongside quantitative analysis of contrast-to-noise ratio (CNR) and edge rise distance (ERD). Surgical pathology correlation was available for 33 cases. Five radiologists independently reviewed the images.

**Results:**

Among 153 matched examination pairs from 132 patients (mean age, 58 ± 11 years), AI-MRI outperformed CECT in both qualitative and quantitative measures. MRI yielded higher image quality scores (4 [IQR 3–4] vs. 3 [IQR 3–3]), improved CNR (5.5 [IQR 4.2–7.0] vs. 1.3 [IQR 0.7–2.2]), and reduced ERD (1.6 mm [IQR 1.2–2.8] vs. 3.7 mm [IQR 2.8–5.0]). MRI provided superior peritoneal sharpness (75.8%) and colonic wall visualization (100%), and superior or equivalent tumor conspicuity (92.2%) and nodal assessment (86.9%), whereas CECT demonstrated better artifact control (45.1%). In preliminary analyses, MRI achieved higher T-staging accuracy (78.8% vs. 30.3%) and N-staging specificity (93.3% vs. 13.3%).

**Conclusion:**

The AI-accelerated MRI protocol significantly outperformed CECT in image quality and preliminary staging accuracy for colon cancer evaluation while eliminating radiation exposure.

**Key Points:**

***Question*** Can an AI-accelerated MRI protocol provide superior image quality and staging performance compared with contrast-enhanced CT, which has limited diagnostic accuracy for colon cancer staging?

***Findings*** AI-accelerated MRI using L₁-regularized compressed sensing and deep learning reconstruction achieved superior image quality and higher preliminary staging accuracy than contrast-enhanced CT.

***Critical Relevance Statement*** AI-accelerated MRI improves image quality and staging confidence without radiation exposure, supporting its potential to enhance preoperative assessment and clinical decision-making in colon cancer.

**Graphical Abstract:**

**Figure:**
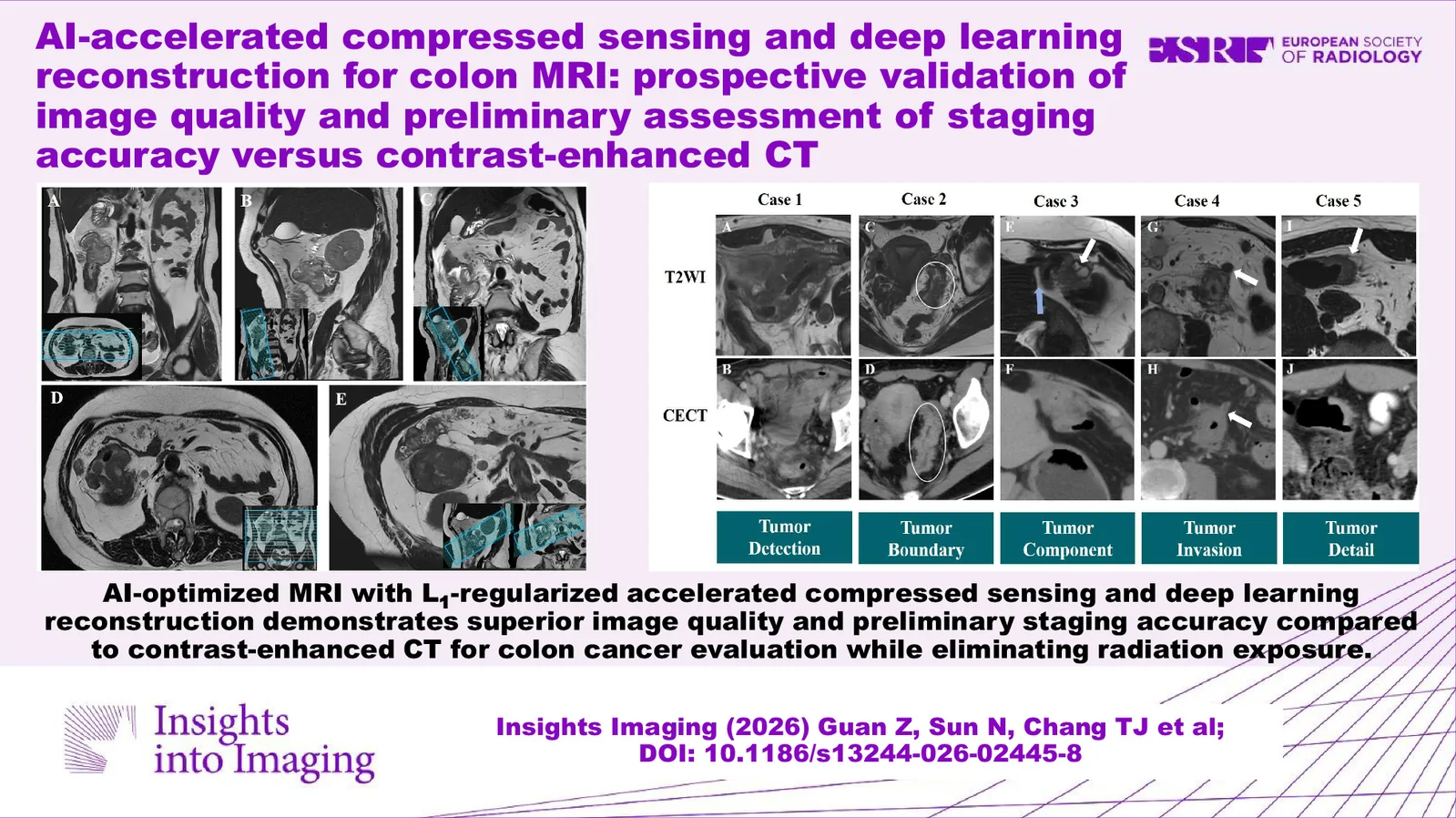

## Introduction

Colorectal cancer (CRC) is the fourth most common cancer and the second leading cause of cancer-related deaths worldwide, making accurate preoperative staging essential for treatment planning [1–3]. Although contrast-enhanced CT (CECT) remains the standard imaging modality for colon cancer evaluation, its limited soft-tissue contrast constrains staging accuracy. Previous studies have reported high sensitivity (90–95%) but only moderate specificity (50–69%) for detecting tumor invasion beyond the bowel wall (T3–T4), along with moderate performance for nodal staging, where sensitivities and specificities generally range from 41% to 71%. Interobserver variability further compounds these limitations [4–7]. Moreover, CECT involves risks of radiation exposure and contrast-related adverse reactions.

MRI is considered the gold standard for rectal cancer staging owing to its superior soft-tissue contrast; however, its application in colon cancer remains limited by technical challenges [8–10]. The primary limitation arises from motion artifacts caused by both intestinal peristalsis and respiratory motion. In addition, the need for high spatial resolution necessitates longer acquisition times. Respiratory-triggered T2-weighted imaging (T2WI) is currently the only feasible approach for colon MRI, yet it often yields variable image quality. Furthermore, the considerable anatomical variability of colonic segments poses additional challenges for transverse imaging of colonic lesions. Collectively, these factors restrict the routine clinical use of MRI for colon cancer assessment, underscoring an urgent need for technological advancement in this field.

Artificial intelligence (AI) is transforming MRI through innovative approaches that address long-standing limitations in abdominal imaging. AI-accelerated compressed sensing (ACS) combines k-space undersampling with parallel imaging to enable breath-hold acquisitions. Although ACS has been successfully applied in breast and cardiac imaging [11–13], existing implementations rely on conventional parallel imaging reconstruction and lack dedicated mechanisms for suppressing irregular colonic motility artifacts. Deep learning–based reconstruction (DeepRecon) employs neural networks for dual-domain (k-space and image-space) processing to simultaneously enhance signal recovery and suppress noise, as validated in liver and prostate imaging; however, its application to colonic imaging remains unexplored [14–16]. To specifically address the unique challenges of colon MRI, we incorporated L₁-norm regularization into the ACS framework. By enforcing sparsity in the total variation domain during iterative reconstruction, the integrated L₁-regularized ACS framework achieves generalized discrimination between true anatomical structures and motion artifacts without requiring motion-specific priors [17, 18]. Theoretical integration with the noise suppression capability of DeepRecon suggests that this combined strategy could provide both motion robustness and preserved spatial resolution.

Therefore, this study aimed to develop and validate an AI-accelerated MRI protocol integrating L₁-regularized ACS and DeepRecon, compare its image quality with that of CECT, and perform a preliminary assessment of clinical staging accuracy.

## Materials and methods

### Study participants

**Fig. 1:**
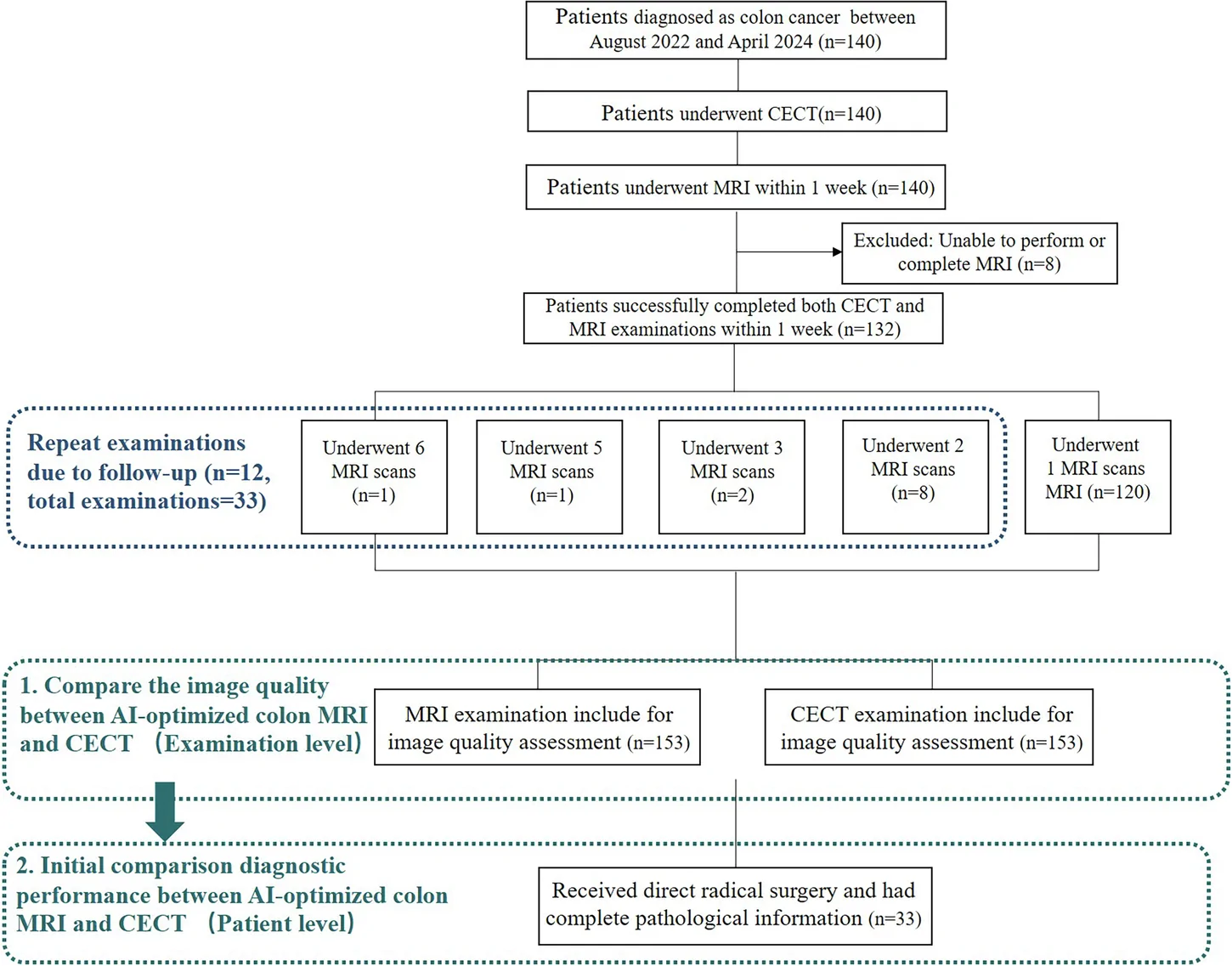
Study inclusion and exclusion flowchart. CECT, contrast-enhanced CT

This prospective, single-center study was approved by the Institutional Review Board of Peking University Cancer Hospital (2022YJZ68). Participation was voluntary, and written informed consent was obtained from all patients. Consecutive patients with colon cancer were enrolled between August 2022 and April 2024. The inclusion criteria were as follows: (1) colon cancer confirmed by colonoscopic biopsy and (2) non-contrast colon MRI and CECT performed within 1 week. The exclusion criteria were as follows: (1) contraindications to MRI (e.g., non-MRI-compatible implants or severe ferromagnetic artifacts from prior surgery) and (2) inability to breath-hold or claustrophobia preventing completion of MRI (Fig. 1).

### MRI preparation and protocol

MRI was performed using a 3.0-T scanner (uMR790, United Imaging Healthcare) with a SuperFlex coil, with patients in the supine position. After fasting for 4–6 h, patients without contraindications received 20 mg of intramuscular scopolamine butylbromide 20 min before scanning to minimize peristalsis. No antiperistaltic agents were administered to patients with contraindications.

**Fig. 2:**
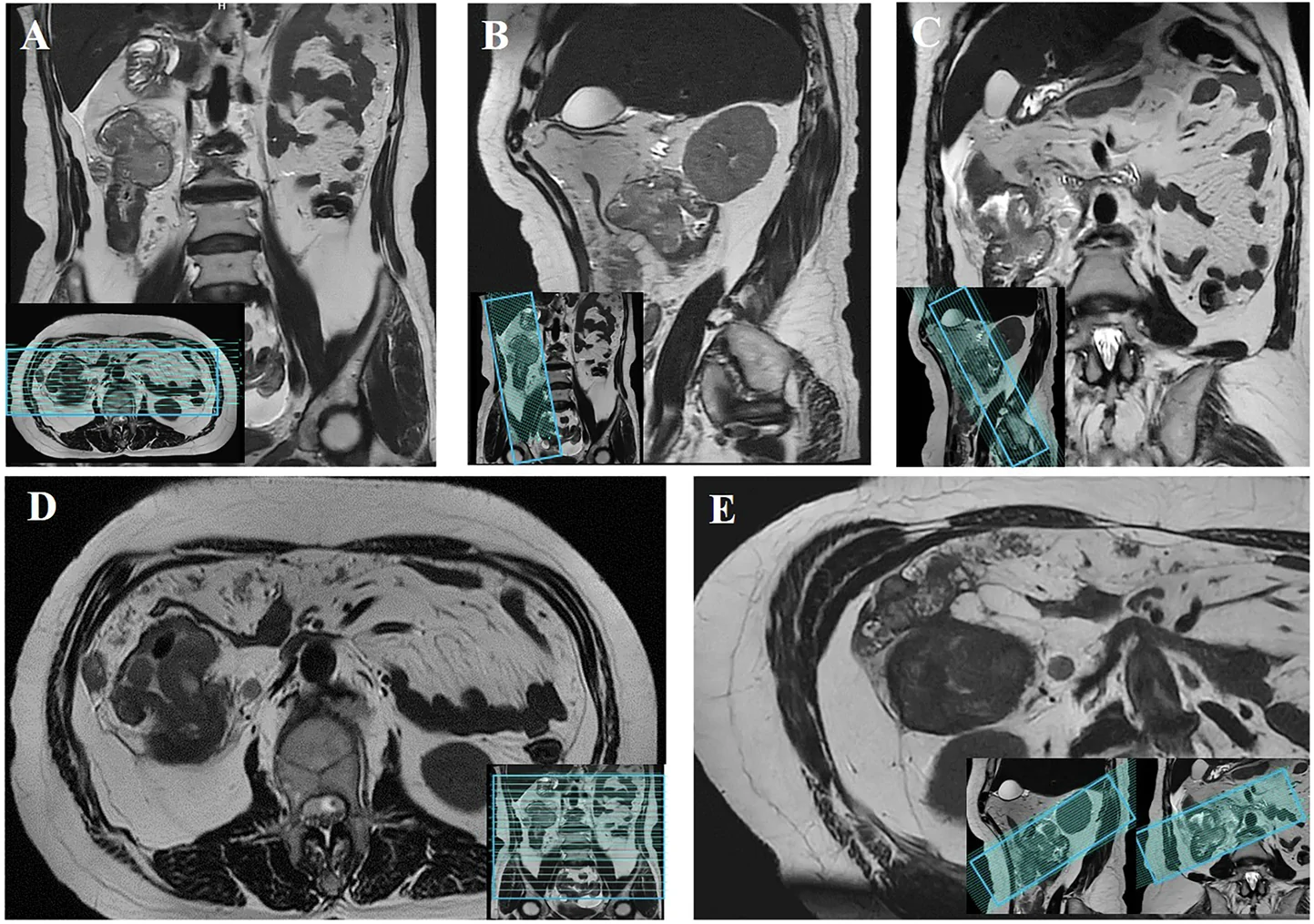
Scanning order and coverage of different T2WI sequences. **A** Coronal T2WI. **B** Oblique sagittal T2WI. **C** Oblique coronal T2WI. **D** Axial T2WI. **E** Oblique axial T2WI. T2WI, T2-weighted imaging

The imaging protocol incorporated tailored tri-oblique planes to account for anatomical variations along the colonic segments and optimize tumor delineation (Fig. 2). The standardized MRI protocol comprised the following sequences: (1) Transverse T2WI with tri-oblique planes: the first oblique plane was parallel to the long axis of the lesion on the coronal image, the second oblique plane was parallel to the long axis of the lesion on the first oblique image, and the transverse plane was perpendicular to the long axis of the lesion on both oblique images. In cases of tortuous anatomy, the colon was divided into discrete segments, with individualized tri-oblique plane positioning for each segment. (2) Axial diffusion-weighted imaging (DWI) acquired at *b*-values of 50 and 1000 s/mm². (3) Axial T1-weighted imaging (T1WI) and T2WI. Both T2WI and T1WI sequences were acquired during breath-hold conditions. Comprehensive technical parameters for all sequences are provided in Tables 1 and 2. The total MRI acquisition time was approximately 20 min, varying slightly depending on patient cooperation and lesion extent.

**Table 1:** MRI protocol for colon

Items	Details
Magnet strength	3.0 T
Coil	SuperFlex Coil
Patient preparation	
Fasting	4–6 h
Antiperistaltic agent	Scopolamine butylbromide (patients with contraindications were not injected)
Bowel preparation	None
Urinary bladder filling	Poor
Sequences	
T2-weighted fast (turbo) spin-echo	Coronal Section thickness: 4 mm Coverage: the dome of the diaphragm to the inferior pelvic margin
T2-weighted fast (turbo) spin-echo	First oblique (parallel to the long axis of the lesion seen on the coronal image) Section thickness: 3 mm Coverage: includes the lesion
T2-weighted fast (turbo) spin-echo	Second oblique (parallel to the long axis of the lesion seen on the first oblique image) Section thickness: 3 mm Coverage: includes the lesion
T2-weighted fast (turbo) spin-echo	Transverse (perpendicular to the long axis of the lesion scan seen on first and second oblique images), small field of view Section thickness: 3 mm Coverage: include the lesion
T2-weighted fast (turbo) spin-echo	Axial, large field of view Section thickness: 5 mm Coverage: the dome of the diaphragm to the inferior pelvic margin
Diffusion-weighted	Axial: *b* = 50, 1000 s/mm^2^ Coverage: include the lesion
T1-weighted fat saturation	Axial, large field of view
T1 in-phase and out-of-phase Dixon	Axial, large field of view

**Table 2:** Parameters for AI-accelerated colon MRI

Parameter	Coronal T2WI	Oblique Sagittal T2WI	Oblique Coronal T2WI	Axial T2WI	Oblique Axial T2WI	Axial T1WI	DWI (*b* = 1000 s/mm^2^)
TR (ms)	9414	7527	7696	4100	3217	4.56	3750
TE (ms)	110	110	108	108	115	1.65	49.2
Voxel size (mm^3^)	1.1 × 1.1 × 4.0	1.1 × 1.1 × 4.0	1.1 × 1.1 × 4.0	1.0 × 0.9 × 5.0	0.73 × 0.73 × 3.0	1.4 × 1.2 × 4.4	2.5 × 2.5 × 5.0
Field of view (mm^2^)	400 × 350	350 × 320	350 × 320	350 × 320	280 × 280	350 × 300	320 × 320
Matrix size	320 × 320	320 × 320	320 × 320	352 × 282	384 × 384	288 × 222	128 × 128
Refocus Flip angle (°)	120	120	120	150	140	12	--
Bandwidth (Hz/Px)	450	450	450	500	400	800	2600
Number of Excitations	1	1	1	1	1	1	8
Section thickness (mm)	4.0	3.0	3.0	5.0	3.0	4.0	5.0
Gap (mm)	0.8	0.8	0.8	1.0	0.3	50% overlap	1.0
Slices	30–40	10	10	40–60	20–24	80	40–60
Scan time (min:s)	0:15	0:10	0:10	0:45	0:30	0:15	5:30

*TE* echo time, *TR* repetition time

Plane prescription was primarily performed by trained MRI technologists according to predefined anatomical rules. Before study initiation, all technologists underwent protocol-specific training to standardize lesion-oriented plane planning. When lesion orientation was unclear, consultation with an attending radiologist was obtained.

### L₁-regularized ACS reconstruction for motion artifact suppression

**Fig. 3:**
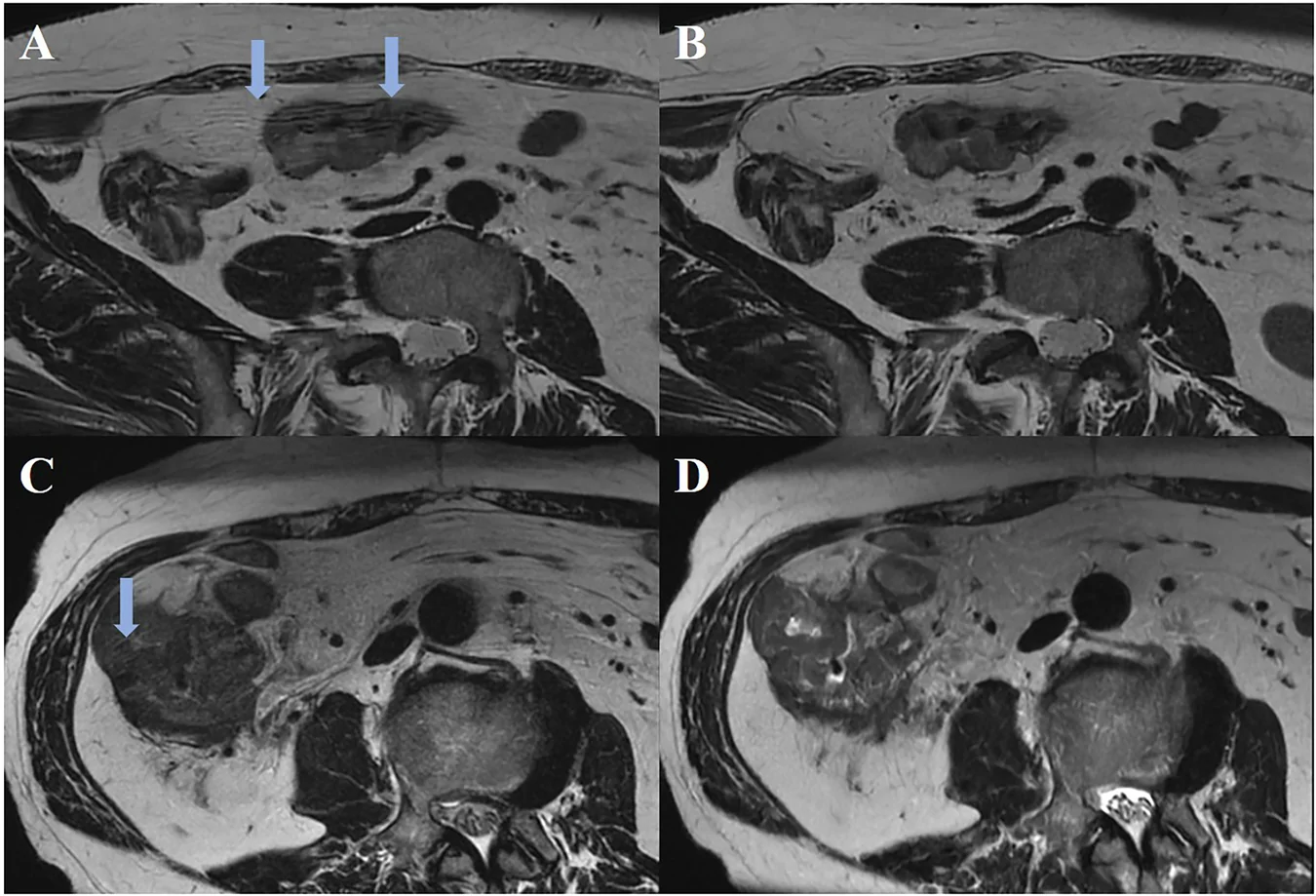
Representative MRI images without and with L₁-norm regularization. Sigmoid colon adenocarcinoma in a 37-year-old man (**A**, **B**) and ascending colon adenocarcinoma in a 45-year-old woman (**C**, **D**). Oblique axial T2WI without L₁-norm regularization demonstrates parallel imaging artifacts (blue arrows) (**A**, **C**). Oblique axial T2WI with L₁-norm regularization shows improved image quality with artifact reduction and better tumor delineation (**B**, **D**). T2WI, T2-weighted imaging

To mitigate motion artifacts, L₁-norm regularization was incorporated into the ACS reconstruction framework. This approach enforces sparsity by minimizing the L₁ norm of the coefficients in the total variation domain, which compresses low-amplitude components (e.g., noise and artifacts) toward zero while preserving high-magnitude anatomical structures. The iterative soft-thresholding algorithm (ISTA/FISTA) was employed to apply soft-thresholding to sparse coefficients, thereby eliminating low-amplitude noise and artifact components. Vendor-provided algorithm parameters were used. Representative images comparing the effects of L₁-norm regularization are presented in Fig. 3.

### CECT preparation and protocol

CT examinations were performed using either a SOMATOM Force or a Revolution CT scanner. After fasting for 4–6 h, patients were instructed to drink 500 mL of water 20 min before the examination. Patients were then scanned in the supine position to obtain unenhanced images. A nonionic contrast agent (Ultravist 300, Schering, or Omnipaque 300, Nycomed) was injected at a rate of 3 mL/s with a dose of 2 mL/kg adjusted according to body weight. Arterial, venous, and delayed phase images were acquired at 25–40 s, 70–80 s, and 150 s after injection, respectively. Venous phase images were transferred to an AW4.2 workstation for coronal and sagittal reconstruction. The scanning parameters were as follows: tube voltage, 120 kV; tube current, 100–300 mA; rotation time, 0.8 s; detector collimation, 0.625 mm; pitch, 0.983; and slice thickness and interval, 5 mm.

### Qualitative image analysis

Five radiologists with varying levels of experience (junior radiologists with 9 and 7 years of experience, respectively; in-training radiologists with 3, 2, and 1 years of experience, respectively) independently assessed the entire image set according to a predefined consensus protocol, and interobserver agreement was recorded. An experienced reader (with 30 years of experience in abdominal radiology) provided the final adjudication in cases of disagreement.

Peritoneal and colonic wall sharpness, tumor and lymph node visibility, and artifacts were graded using a modified 4-point Likert scale to determine composite image quality (Table S1). The total score across all evaluation items determined the final image quality grade: 5–8 points, grade 1 (poor); 9–12 points, grade 2 (moderate); 13–16 points, grade 3 (good); and 17–20 points, grade 4 (excellent).

### Quantitative image analysis

The contrast-to-noise ratio (CNR) was calculated by manually delineating two regions of interest (ROIs): tumor and muscle. The tumor ROI was placed within the delineated tumor area on a predefined axial slice showing the largest tumor cross-sectional diameter. The muscle ROI was placed in the corresponding muscle on the same slice, avoiding visible fat infiltration, vessels, and motion artifacts. Both tumor and muscle ROIs were manually drawn with an equal area (25 mm²) and were kept consistent in location and size across repetitions. All ROI delineations followed the same protocol for each modality. CNR was defined as the ratio of the difference in mean signal intensity between the tumor and muscle to the square root of the sum of the variances of the tumor and muscle signal intensities [19]. Image sharpness was quantified using the edge rise distance (ERD), defined as the distance between the 10% and 90% points of the maximum intensity value [20]. CNR and ERD measurements were repeated three times by the same reader on the same images. For each case, the final CNR and ERD values were calculated as the mean of the three repeated measurements. Measurements were performed on axial T2-weighted images for MRI and on venous-phase images for CECT (Fig. S1).

### Image evaluation

The T-stage evaluation criteria were defined as follows: T1, tumor invades the submucosa without reaching the muscularis propria; T2, tumor extends into but does not penetrate the muscularis propria; T3, tumor invades beyond the muscularis propria into pericolonic fat; T4a, tumor penetrates the visceral peritoneal surface; and T4b, tumor directly invades adjacent organs or structures. Evaluations were performed on venous-phase CT and oblique axial T2-weighted MRI. Lymph nodes were considered metastatic if they measured ≥ 10 mm in short-axis diameter or ≥ 5 mm with irregular borders and/or heterogeneous signal intensity/density [21]. Nodal staging was simplified to a binary system: N− (no metastatic nodes) or N+ (metastatic nodes present). Coronal and sagittal images were reviewed when necessary to further evaluate tumor extent and involvement of adjacent structures. T/N staging assessments were conducted in two independent reading sessions (CT-first, followed by MRI) separated by a 4-week washout period. Because CT and MRI are inherently distinguishable, complete blinding to modality was not feasible; however, five radiologists independently reviewed all images while blinded to pathological results and clinical information, with disagreements resolved by consensus for the final analysis.

### Statistical analysis

Normality was assessed using the Kolmogorov-Smirnov test. Categorical variables were expressed as absolute numbers and percentages. Normally distributed continuous variables were reported as means and standard deviations (SDs), whereas non-normally distributed variables were presented as medians and interquartile ranges (IQRs). Paired comparisons of qualitative and quantitative image quality between MRI and CECT within the same participant were performed using Wilcoxon signed-rank tests. For all paired comparisons, effect sizes were reported as paired differences with 95% confidence intervals (CIs). Interobserver agreement was assessed using pairwise Cohen’s *κ* statistics, with corresponding 95% CIs (< 0.20, poor; 0.21–0.40, fair; 0.41–0.60, moderate; 0.61–0.80, substantial; 0.81–1.00, excellent). Agreement between imaging-based T staging and pathology was assessed using quadratic weighted kappa statistics. McNemar’s test was used to compare paired diagnostic performance measures between MRI and CECT. A *p*-value < 0.05 was considered statistically significant. All statistical analyses were conducted using SPSS software (version 26).

## Results

### Participant characteristics

Of the 140 patients, 8 were excluded due to inability to maintain breath-holding or claustrophobia preventing MRI completion (Fig. 1). Colon MRI was successfully performed in 132 of 140 participants (94.3%), with a mean acquisition time of approximately 20 min. The mean age was 58.0 ± 11.0 years (range, 29–79 years), and the cohort included 79 males and 53 females. Among these participants, 120 underwent a single examination, and 12 underwent multiple follow-up examinations; the final analysis comprised 153 matched MRI and CECT examinations from these 132 participants. Additionally, 33 consecutive participants who underwent direct radical surgery with complete pathological data were included. The interval between colon MRI and surgery was less than 2 weeks. Demographic and clinicopathological characteristics of participants with and without surgical pathology, including sex, age, tumor location, and histology type, are summarized in Table 3. Only histology type differed significantly between the two groups (*p* = 0.014); no significant differences were observed in sex, age, or tumor location.

**Table 3:** Comparison between patients with surgical pathology and those without surgical pathology

Characteristic	Full cohort (*n* = 132)	Pathology-correlated subset (*n* = 33)	Patients without surgical pathology (*n* = 99)	*p*
Sex				0.124
Male	79 (60)	16 (48)	63 (64)	
Female	53 (40)	17 (52)	36 (36)	
Mean age (years)	58 ± 11	60 ± 11	58 ± 11	0.123
Tumor location				0.404
Ascending	22 (17)	6 (18)	16 (16)	
Hepatic flexure	23 (17)	3 (9)	20 (20)	
Transverse	12 (9)	3 (9)	9 (9)	
Splenic flexure	4 (3)	1 (3)	3 (3)	
Descending	13(10)	5 (15)	8 (8)	
Sigmoid	58 (44)	15 (46)	43 (44)	
Histology type				0.014
Non-mucinous carcinoma	127 (96)	29 (88)	98 (99)	
Mucinous carcinoma	5 (4)	4 (12)	1 (1)	
pT^a^				/
T1		0 (0)	/	
T2		2 (6)	/	
T3		25 (76)	/	
T4a		3 (9)	/	
T4b		3 (9)	/	
pN^a^				/
N0		15 (46)	/	
N1		12 (36)	/	
N2		6 (18)	/	

Unless otherwise indicated, data are numbers of participants; data in parentheses are percentages. Mean data are ± SDs, with ranges in parentheses

*pN* pathologic lymph node stage, *pT* pathologic tumor stage

^a^ Two pathological characteristics (pT and pN stages) were analyzed in the cohort of patients who underwent direct surgery and had complete pathological data

### Qualitative image analysis between AI-accelerated MRI and CECT

AI-accelerated MRI demonstrated superior performance across multiple image quality metrics compared with CECT. Peritoneal sharpness was rated higher in 75.8% (116/153) of MRI examinations, with the remaining 24.2% (37/153) rated as equivalent (median, 3 [IQR, 3–4] vs. median, 2 [IQR, 1–3]; *p* < 0.001). Colonic wall visualization was better on MRI in all examinations. Tumor conspicuity was superior or equivalent in 92.2% (141/153) of MRI cases (median, 4 [IQR, 3–4] vs. median, 3 [IQR, 3–4]; *p* < 0.001), and lymph node assessment was improved or equivalent in 86.9% (133/153) of examinations (median, 3 [IQR, 3–4] vs. median, 3 [IQR, 2–3]; *p* < 0.001). CECT showed better artifact control in 45.1% (69/153) of cases (median, 4 [IQR, 4–4] vs. median, 4 [IQR, 3–4]; *p* < 0.001). MRI achieved a higher overall image quality grade in 60.8% (93/153) of examinations (median, 4 [IQR, 3–4] vs. median, 3 [IQR, 3–3]; *p* < 0.001). Pairwise interobserver agreement among the five readers ranged from moderate to excellent (*κ* = 0.45–1.00), with corresponding 95% CIs ranging from 0.32–0.61 to 0.92–1.00. For CECT artifact assessment, perfect agreement was observed among readers (*κ* = 1.00; 95% CI estimated using bootstrap). For CECT bowel wall sharpness, most ratings clustered in a single category (median, 1 [IQR, 1–1]), which may have influenced the kappa value due to the prevalence paradox associated with highly skewed distributions; nevertheless, the observed percentage agreement among readers remained high (91.2%). Detailed image quality assessments are provided in Tables 4 and 5.

**Table 4:** The proportion of comparisons between AI-accelerated MRI and CECT for the image quality assessment

Parameter	MRI > CECT	MRI = CECT	MRI < CECT
Overall image quality	93 (60.8)	45 (29.4)	15 (9.8)
Sharpness of the peritoneum	116 (75.8)	37 (24.2)	0 (0)
Sharpness of bowel wall layers	153 (100)	0 (0)	0 (0)
Tumor conspicuity	77 (50.3)	64 (41.8)	12 (7.9)
Lymph node conspicuity	71 (46.4)	62 (40.5)	20 (13.1)
Artifacts	5 (3.3)	79 (51.6)	69 (45.1)

Data in parentheses are percentages

*CECT* contrast-enhanced CT

**Table 5:** Image quality assessment between AI-accelerated MRI and CECT in colon cancer

Parameter	MRI	*κ*-value (95% CI)	CECT	*κ*-value (95% CI)	*p-*value
Image quality scores					
Overall image quality	4 (3–4)	0.512 (0.38–0.65)	3 (3–3)	0.452 (0.32–0.61)	< 0.001
Sharpness of the peritoneum	3 (3–4)	0.576 (0.45–0.71)	2 (1–3)	0.448 (0.31–0.59)	< 0.001
Sharpness of bowel wall layers	4 (4–4)	0.492 (0.35–0.63)	1 (1–1)	0.498 (0.36–0.64)	< 0.001
Lesion conspicuity	4 (3–4)	0.601 (0.47–0.73)	3 (3–4)	0.475 (0.34–0.61)	< 0.001
Lymph node conspicuity	3 (3–4)	0.586 (0.46–0.72)	3 (2–3)	0.479 (0.34–0.62)	< 0.001
Artifacts	4 (3–4)	0.746 (0.64–0.85)	4 (4–4)	1.000 (0.92–1.00)	< .001
CNR	5.5 (4.2–7.0)	…	1.3 (0.7–2.2)	…	< 0.001
ERD	1.6 (1.2–2.8)	…	3.7 (2.8–5.0)	…	< 0.001

Data are medians; data in parentheses are IQRs. Data were compared using the Wilcoxon signed-rank tests

*CNR* contrast-to-noise ratio, *CI* confidence interval, *ERD* edge rise distance

### Quantitative image analysis between AI-accelerated MRI and CECT

**Fig. 4:**
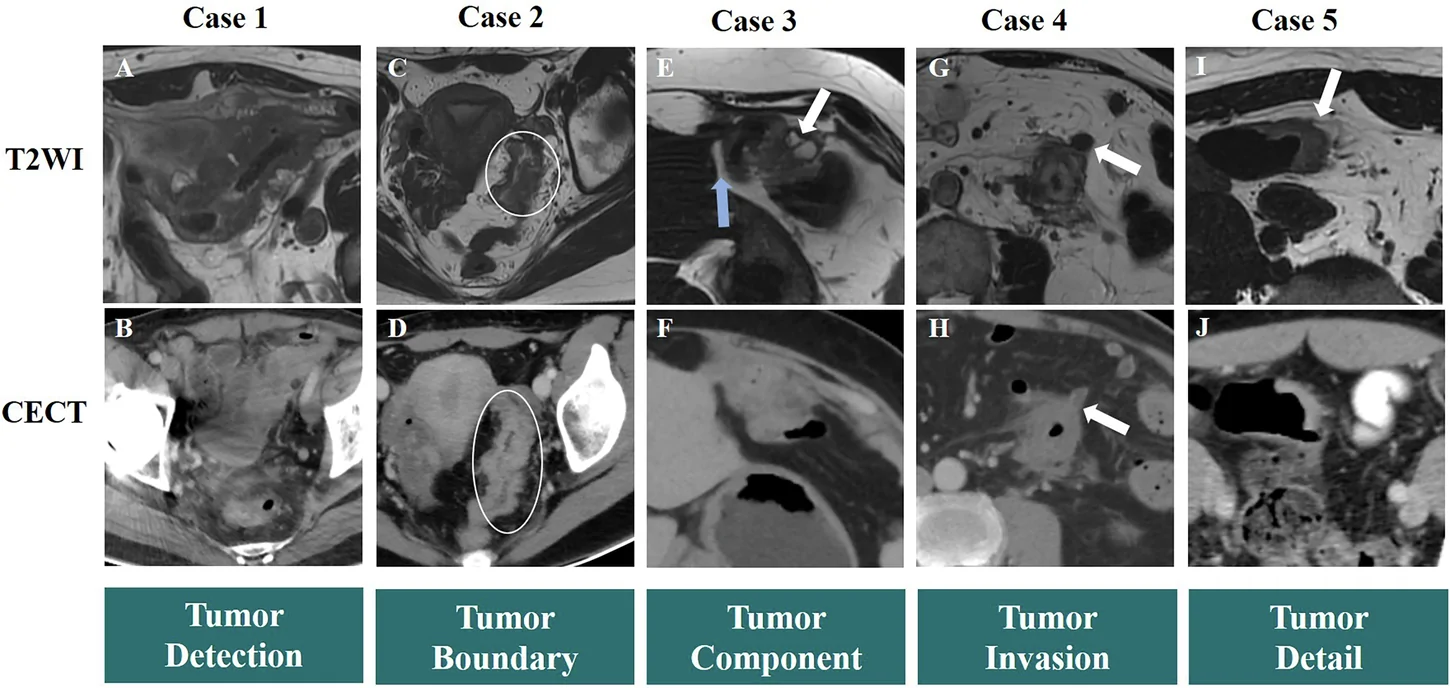
Representative images of AI-accelerated MRI and CECT in colon cancer. **A**, **B** Sigmoid colon adenocarcinoma in a 73-year-old man after femoral head replacement: **A** T2WI demonstrates preserved visualization of key anatomic structures, including the peritoneum, primary tumor, and suspicious lymph nodes, unaffected by metallic implants. **B** CECT fails to demonstrate these structures owing to metal artifacts. **C**, **D** Sigmoid colon adenocarcinoma in a 41-year-old woman: **C** T2WI clearly delineates the lesion extent and its boundaries (circle). **D** CECT reveals a longer segment of thickened bowel wall (circle) due to peristaltic motion, obscuring the tumor–normal tissue interface. **E**, **F** Transverse colon mucinous carcinoma in a 60-year-old man: T2WI (**E**) reveals a distinct mucinous component (white arrow), which is difficult to recognize on CECT (**F**). Notably, although subtle respiratory motion artifacts (blue arrow) are observed on T2WI due to the subperitoneal location of the lesion, they do not compromise diagnostic interpretation; in fact, the image quality remains superior to that of an artifact-free CECT scan. **G**, **H** Descending colon adenocarcinoma in a 55-year-old woman: **G** T2WI demonstrates tumor proximity to morphologically intact small bowel (white arrow), staged as cT4a. **H** CECT suggests tumor invasion into adjacent small bowel (white arrow), interpreted as clinical T4b. Final pathological staging confirmed pT4a (no small bowel invasion). **I**, **J** Sigmoid colon adenocarcinoma with synchronous liver metastasis in a 29-year-old man: **I** T2WI clearly demonstrates extramural vascular invasion (white arrow), a high-risk feature. **J** CECT fails to identify any high-risk imaging characteristics. CECT, contrast-enhanced CT; T2WI, T2-weighted imaging

The CNR was significantly higher for MRI than for CECT (5.5 [IQR, 4.2–7.0] vs. 1.3 [IQR, 0.7–2.2]; *p* < 0.001). The medium ERD was significantly lower for MRI than for CECT (1.6 mm [IQR, 1.2–2.8] vs. 3.7 mm [IQR, 2.8–5.0]; *p* < 0.001) (Table 5). Representative images are shown in Fig. 4. In a sensitivity analysis restricted to the first examination per patient (*n* = 132), CNR and ERD values were nearly identical to those in the full dataset (*p* > 0.05). At the patient level, MRI remained significantly superior to CECT for both CNR (5.4 [IQR, 4.3–6.9] vs. 1.4 [IQR, 0.8–2.2]; *p* < 0.001) and ERD (1.7 mm [IQR, 1.2–2.8] vs. 3.6 mm [IQR, 2.8–5.0]; *p* < 0.001).

### Preliminary comparison of T and N staging between AI-accelerated MRI and CECT

For tumor (T) staging based on T2-weighted MRI, the accuracy was 78.8% (26/33; 95% CI: 62.0, 89.6), with overstaging and understaging rates of 18.2% (6/33; 95% CI: 8.2, 34.8) and 3.0% (1/33; 95% CI: < 0.01, 16.7), respectively. For CECT, the accuracy was 30.3% (10/33; 95% CI: 17.3, 47.3), with overstaging in 60.6% (20/33; 95% CI: 43.7, 75.4) and understaging in 9.1% (3/33; 95% CI: 2.4, 24.3) of cases (Table S2). MRI demonstrated substantial agreement with pathology for T staging (*κ* = 0.79; 95% CI: 0.55–0.93), whereas CECT showed only fair agreement (*κ* = 0.27; 95% CI: 0.02–0.55).

For lymph node (N) staging, T2WI achieved an accuracy of 81.8% (27/33; 95% CI: 65.2, 91.8), a sensitivity of 72.2% (13/18; 95% CI: 46.4, 89.3), and a specificity of 93.3% (14/15; 95% CI: 66.0, 99.7). In contrast, CECT achieved an accuracy of 51.5% (17/33; 95% CI: 35.2, 67.5), a sensitivity of 83.3% (15/18; 95% CI: 57.7, 95.6), and a specificity of 13.3% (2/15; 95% CI: 2.3, 41.6). MRI demonstrated significantly higher specificity than CECT (*p* < 0.001).

## Discussion

Accurate preoperative staging is essential for surgical planning and neoadjuvant therapy selection in colon cancer [22, 23]. Although MRI provides superior soft-tissue contrast without ionizing radiation, its application in colon imaging remains technically challenging. Our study demonstrates that an AI-accelerated MRI protocol combining an L₁-regularized ACS framework and DeepRecon technology with tri-oblique plane acquisitions significantly outperforms CECT, yielding higher median image quality scores (4 [IQR, 3–4] vs. 3 [IQR, 3–3]; *p* < 0.001), improved CNR (5.5 vs. 1.3; *p* < 0.001), and better edge sharpness (ERD, 1.6 mm vs. 3.7 mm; *p* < 0.001). This approach addresses longstanding limitations of colon MRI, namely the speed-quality tradeoff and susceptibility to motion artifacts, while preserving diagnostic accuracy.

The primary challenge in colon MRI is effective motion suppression. Although breath-holding remains the optimal strategy, high-resolution protocols inevitably prolong acquisition time. Parallel imaging with high acceleration factors can reduce scan duration but introduces parallel acquisition artifacts; even minor breath-holding inconsistencies can generate substantial artifacts. Previously reported colon MRI techniques have been limited to either respiratory-triggered sequences or single-shot FSE breath-hold protocols, both of which result in inconsistent image quality and motion artifacts. Although prior studies have suggested a potential role for MRI in colon cancer staging, these limitations have constrained its clinical reliability and broader adoption [8–10].

Our study demonstrates that the AI-accelerated MRI protocol integrating L₁-regularized ACS and DeepRecon represents a substantial advancement in colon imaging by combining accelerated acquisition with superior image quality. Specifically, the accelerated sequence parameters (repetition time > 2500 ms) enabled acquisition of 24 high-resolution slices in 30 s using two breath-holds, achieving a 50% reduction in both scan time (vs. conventional 60-s protocols) and breath-hold frequency (vs. four breath-holds) while preserving diagnostic image quality. The integration of L₁ regularization demonstrated substantial artifact suppression in ACS-reconstructed images. By promoting sparsity in the total variation domain, this reconstruction framework reliably preserves anatomical features while suppressing motion-induced artifacts, including high-frequency noise and structured ghosting. Notably, the integrated L₁-ACS framework provides generalized robustness against motion artifacts, eliminating the need for prior knowledge of specific motion patterns and delivering consistent performance across diverse patient conditions, as validated in this study. Such capability is critical for breath-hold imaging and motion-prone examinations, where traditional methods often fail [17, 18]. The artifacts observed on MRI were predominantly mild residual motion, and the reported artifact reduction reflects improvement relative to conventional ACS-based MRI rather than superiority over CECT, without compromising diagnostic confidence.

DeepRecon is a proprietary deep learning reconstruction model (United Imaging Healthcare) that is fine-tuned for abdominal imaging prior to clinical use. It employs a dual-domain (k-space and image-space) neural network to enhance signal recovery and suppress noise [24–26]. Similar approaches have been validated in liver and prostate MRI, although their application to colonic imaging has not been previously reported [14–16]. Detailed architectural specifications remain proprietary. When combined with L₁-regularized ACS accelerated acquisition, this approach outperformed CECT, demonstrating a 4.2-fold higher CNR (5.5 vs. 1.3) and a 56% reduction in ERD (1.6 mm vs. 3.7 mm), which resulted in improved tumor conspicuity and margin delineation. Although the primary analysis was examination-based, a patient-level sensitivity analysis yielded consistent results, indicating no material impact of within-patient correlation. This accelerated protocol enables 30-s breath-hold imaging while maintaining high image quality (CNR > 5.0) and spatial resolution (0.7 × 0.7 × 3 mm acquired; 0.5 × 0.5 × 3 mm reconstructed), thereby mitigating traditional speed-quality tradeoffs. No evident over-smoothing or artificial texture was observed, and fine anatomical details were preserved without compromising diagnostic confidence. These findings were supported by structured qualitative image quality assessment and are exemplified in Fig. 3B, D.

The tri-oblique scanning protocol, inspired by four-chamber view localization in cardiac MRI, effectively addresses key limitations of colon MRI by minimizing partial volume artifacts while preserving true anatomical relationships, particularly in the transverse and sigmoid colon. Although quantitative artifact measurements were slightly higher for MRI than for CECT (a known strength of CT), the tri-oblique thin-slice (3-mm) high-resolution protocol maintained superior cross-sectional anatomical clarity, with residual artifacts having no clinically substantial impact on lesion detection or overall diagnostic image quality. The additional planning time required for tri-oblique positioning was minimal (approximately 1–2 min) and did not substantially prolong total examination time. Furthermore, comprehensive axial T2WI coverage from the diaphragmatic dome to the inferior pelvic margin enables precise tumor localization and preliminary evaluation of regional and retroperitoneal lymph nodes. This protocol demonstrates particular value for initial assessment of colon cancer by providing complete local tumor staging and evaluation of distant metastasis without ionizing radiation, suggesting its potential as a safer alternative to CECT in selected clinical scenarios.

The bowel-preparation-free design of this protocol preserves native intestinal motility, avoids artificial irritation, and ensures optimal magnetic field homogeneity during scanning. Potential practical factors that may degrade image quality include suboptimal breath-holding, bowel peristalsis, and susceptibility artifacts related to intraluminal gas. In our cohort, standardized breathing instructions were provided before scanning, which minimized motion-related degradation. Although bowel motion and gas-related susceptibility artifacts were theoretically anticipated, they rarely interfered with tumor evaluation in clinical practice.

Antiperistaltic agents administered before scanning can effectively reduce intestinal motility. Our clinical experience indicates that for patients with contraindications (e.g., prostatic hyperplasia or glaucoma), the absence of antiperistaltic medication does not significantly compromise image quality when using AI-accelerated MRI. Given the small subgroup of patients who did not receive antiperistaltic agents, comparative analyses were underpowered and therefore not performed. Future studies with balanced cohorts are needed to systematically evaluate the impact of antiperistaltic agents on image quality and further establish the safety profile of this colon MRI protocol.

This study has several limitations. First, it was conducted at a single center, which may limit generalizability. Additionally, the algorithm is vendor-specific and partially proprietary, potentially limiting reproducibility across different MRI platforms. Multicenter studies involving diverse institutions and imaging systems are warranted to validate the robustness and generalizability of these findings. Second, pathological confirmation was available for only 33 of 153 examinations (22%), as most patients underwent neoadjuvant therapy without subsequent surgical resection; therefore, comparative staging results should be interpreted with caution. The apparent CECT over-staging observed in this subset may relate to inflammatory stranding or tumor volume. Furthermore, mucinous histology was relatively enriched in the pathology-correlated subset (12% vs. 4% in the full cohort), which may have further contributed to CECT underperformance, given the known challenges of CT-based T staging in mucinous tumors. However, the limited sample size precludes definitive conclusions. Importantly, this study primarily aimed to evaluate the clinical feasibility of AI-accelerated MRI with tri-oblique acquisition planes rather than to establish definitive staging accuracy. Prospective multicenter studies implementing this protocol are needed to fully validate its diagnostic performance for colon cancer staging.

The AI-accelerated MRI protocol with tri-oblique acquisition represents a clinically feasible approach for colon imaging, providing superior image quality in both anatomical delineation and lesion detection compared with conventional CECT. These improvements support its potential value for comprehensive preoperative evaluation and suggest that this protocol may address certain limitations of current imaging paradigms for colon cancer assessment.

## Supplementary information

ELECTRONIC SUPPLEMENTARY MATERIAL

## Data Availability

The data used or analyzed during the current study are available from the corresponding author upon reasonable request.

## Abbreviations

ACS: AI-accelerated compressed sensing
CECT: Contrast-enhanced CT
CNR: Contrast-to-noise ratio
DeepRecon: Deep learning-based reconstruction
DWI: Diffusion-weighted imaging
ERD: Edge rise distance
pN: Pathologic lymph node stage
pT: Pathologic tumor stage
T1WI: T1-weighted imaging
T2WI: T2-weighted imaging
